# Bundibugyo Ebola Diagnostics and Global Outbreak Preparedness

**DOI:** 10.20411/pai.v11i2.1132

**Published:** 2026-10-27

**Authors:** Alan L. Landay, Michael M. Lederman

**Affiliations:** 1 University of Texas Medical Branch, Galveston, Texas; 2 Case Western Reserve University, Cleveland, Ohio

**Keywords:** Ebola, Bundibugyo Virus, Outbreak Preparedness, Point-of-Care Diagnostics, Global Health

## Abstract

In this interview, Alan L. Landay and Michael M. Lederman speak with Gene Olinger and Thomas Ksiazek about the ongoing Bundibugyo Ebola outbreak and the broader challenges of epidemic preparedness, including how delayed recognition, limited field diagnostics, security concerns, misinformation, and strained international coordination affect the spread of an outbreak. They also discuss the importance of rapid diagnostics; strong epidemiology and contact tracing; reliable, high-containment laboratory networks; and sustained investment in vaccines, therapeutics, and broad-spectrum countermeasures, and emphasize the important role of community trust/local engagement, protection of health care workers, and global collaboration in controlling Ebola outbreaks and preparing for future threats.

## MICHAEL M. LEDERMAN, MD

Welcome to a timely interview with Gene Olinger and Tom Ksiazek. My name is Michael Lederman. I’m the editor-in-chief of *Pathogens and Immunity*. Today, we’ll be discussing the ongoing Ebola virus outbreak. What we have to learn from it, what it means to have epidemic preparedness, and what kind of research infrastructure is needed to be prepared.

## ALAN L. LANDAY, PHD

I’m Alan Landay. I’m a senior editor for *Pathogens and Immunity* and Professor in the Departments of Medicine and Microbiology and Immunology at the University of Texas Medical Branch (UTMB). It’s a great pleasure to introduce two of my colleagues today who will be participating in this discussion on the Ebola outbreak.

First is Dr. Gene Olinger. He is the Director of the Galveston National Laboratory and John Sealy Distinguished University Chair in Tropical and Emerging Virology and Professor in the Department of Microbiology and Immunology at UTMB. He has over 25 years of experience as a research scientist, leading complex studies in biocontainment safety level 3 (BSL-3) and biocontainment safety level 4 (BSL-4) studies. Prior to his appointment at UTMB, he was a Professor at Boston University, Kansas City University, and Hood College. He joined UTMB as Deputy Director of the Galveston National Lab in 2024 and was promoted to Director in 2025.

Our other distinguished guest today is Dr. Tom Ksiazek. He is Director of High-Containment Laboratory Operations at the Galveston National Lab and a world-renowned virus expert with more than 40 years of experience on the front lines of infectious disease research. Through the years, he’s played a significant role in discovery and outbreak responses in Asia, Africa, South America, and the Middle East. He is a Professor in the Department of Pathology and Microbiology and Immunology at UTMB, and he previously served as Chief of the Special Pathogens Branch at the Centers for Disease Control and Prevention (CDC), Atlanta.

Welcome, both Gene and Tom. It’s a real pleasure to have you here today.

First, let’s talk about the big picture. The current Bundibugyo outbreak has drawn global attention. How does this outbreak compare to previous Ebola outbreaks, and what makes it particularly concerning from a scientific and public health perspective? Gene, we’ll start with you and then go to Tom.

## GENE OLINGER, PHD

Thank you again for taking the opportunity to talk about this topic. I’m glad to have Dr. Ksiazek with us, because he’s probably forgotten more than I know, so he’ll be able to correct my answers when I get them wrong.

I think there’s some good news from this outbreak. We have increased capability and capacity, especially in Africa, where Africa CDC now has a larger role. There’s a global network of labs, including high-containment labs. Genomic sequencing now allows us to do things that we couldn’t do before.

International collaborations are somewhat strained at the moment, and it’s more political than it is anything else, but we really have a lot of things going on for the good. The common thing we see in every outbreak is that when you see this level of transmission, something over 500 cases, we start to see features that weren’t known before, including transmission features like walking Ebola, where people are transmitting even when they’re not so sick. Apparently there’s a lot of “walking Ebola.”

We have common and new trust issues locally, nationally, and internationally that we have to manage, including the kind of issues we have now with disengagement from the World Health Organization (WHO). And then there are always funding issues, and those are always new and unique and different.

## THOMAS KSIAZEK, DVM, PHD, MS

The scope of the outbreak is probably the most notable thing, and that’s largely because it wasn’t identified early on, so it spread in an area that was also very challenged because of security issues. I think the second-largest Ebola outbreak occurred in the same area, largely because control efforts were hindered by the security situation. It went on for a couple of years, and that’s playing out to some extent with this one. And then the late recognition of this as an Ebola outbreak led to a wide geographic distribution before people recognized what it was and control efforts began.

## ALL

Tom, you were mentioning the outbreak; how many cases do we have, and how many deaths have we seen to date?

## TK

Well, the last figures I’ve seen were, I think, there are over 2,000 confirmed cases and over 600 deaths at the moment. (Editor’s note: As of October 7, 2026, there were 8,809 confirmed cases and 4,245 confirmed deaths in the Democratic Republic of the Congo [DRC], Kenya, Uganda and France [1, 2]).

## ALL

So this is really the biggest we’ve seen in the field of Ebola outbreaks?

## TK

The West African outbreak is still first; I think this one is actually third at the moment, but it’s rapidly headed toward contending for the largest, I would say, based on the projections and what we’ve seen.

## MML

We’ve seen outbreaks due to the Zaire variant before, but there haven’t been as many outbreaks with this Bundibugyo variant. Apart from or in addition to the occurrence of walking Ebola that Gene mentioned, are there other differences between this variant and the Zaire variant that we should take note of?

## TK

The virus is a little different. I was involved in the first outbreak in 2007. I walked around the town of Bundibugyo in the area where it started in a small clinic. It’s not as lethal as what’s called the Ebola virus now, or the Zaire Ebola virus, so the survivability is probably a little bit higher. I don’t know about the term “walking Ebola.” I think people probably walk around before they get terribly sick, whether they’re transmitting it or just moving to other areas. And then when they really get sick, they begin to transmit it after they’ve walked or somehow transported themselves into a new area. And that’s probably in part why you’re seeing new geographic areas in the Democratic Republic of the Congo (DRC) being enlisted into the outbreak on a pretty regular basis right now.

## GO

As far as the transmission goes, a lot of it is due to the fact that we don’t have diagnostics and people do travel and decide that they don’t have the disease and move. And, of course, it’s easier to move now than it was 20 years ago.

## ALL

So that’s really our next question. We have PCR assays, and they’ve been available to help us. But what about diagnostics for Bundibugyo? I think we heard from Tom and you that we lag behind in identifying the Ebola outbreak because of the lack of diagnostics. Where are we now, and what do we really need for the field going forward?

## TK

I mean, what really helps is having on-site diagnostics in some way. The first outbreaks were in an era before PCR was even available. And so up until the Gulu outbreak, on-site diagnostics were not used. Gulu was really the first one where diagnostics for a couple of types were actually inserted into the center of the outbreak in Gulu, and they helped tremendously. And that’s been pretty much the pattern, whether it’s PCR or antigen detection assays that are used. There are diagnostics for Bundibugyo, but unfortunately early on in this, I guess they either really didn’t consider it or didn’t use those assays that were more poly-Ebola; therefore, I think they sort of dismissed this as not being an Ebola outbreak.

There are certainly other things that, particularly early in the course of the disease, it’s pretty easy clinically to confuse it with. But after the epidemiology reveals itself, it becomes more and more apparent that you have what’s probably an Ebola outbreak on your hands.

## GO

The key thing, like Tom just said, is that PCR is the gold standard these days, but it’s not enough alone. So we need other assays as well. We need to know who was infected, and we need that access as close as we can get to the patient. That’s something we learned from the 2014-15 outbreak. And then the results need to be quick. You can’t have a long period of time. And unfortunately, right now it’s almost impossible to get samples back to Kinshasa, where the national lab is, to run in a reasonable time.

In the early phase of this outbreak, they were using sequencing, which is not realistic either. So getting that testing close to patients is important, but that is a problem in itself, because a lot of the assays, some of the equipment, some of the diagnostic tools that we have, even though they might be miniature, they’re not “ruggedized”. They won’t work as well in hot and humid conditions. So that’s a problem, too.

And often they’re not linked to any epidemiology system other than an Excel spreadsheet. So we really need something different than we have now. We’re working on lateral flow assays (LFA). Some of these, as you saw during COVID-19, can get almost as accurate as PCR, which is one of the reasons people are spending time and effort on them. But we need those assays to be right there with the patient.

And as you’ve seen in the press, too, the families of these patients, because they haven’t been officially diagnosed with an official assay, don’t believe that they’re dead, and they don’t believe they’re dead of that disease. So when they’re burying them. We have a really, really important way of making sure transmission doesn’t happen. But as you’ve seen in *The New York Times* and a couple of other places, the families will surround the burial team and then open up the casket when they leave [3]. So that becomes an issue, because they don’t believe that they’re infected.

## MML

We have a vaccine for the Ebola variant and therapeutics as well, I understand, but nothing yet for Bundibugyo. What does this outbreak teach us about preparedness for rare pathogens that are life-threatening?

## TK

Two outbreaks of Bundibugyo that occurred prior to this one were relatively small and were controlled with tried-and-true methods of case finding and contact tracing. And so people, I guess, have devoted efforts to getting vaccine candidates out there. But following the track to get those to be something that can be used clinically has not advanced greatly. It takes a lot of resources to bring those things to a point where you can start doing clinical trials in the source of an outbreak. Phase 1 and phase 2 studies could be done, but they’re costly, and I guess they weren’t high enough on the priority list to have been done up until now. Now there’s a lot of angst, emphasis, and those things are now all beginning. It’ll take time, though.

## MML

I guess what you’re saying, Tom, is that we are acting in a reactive as opposed to a proactive way. And I understand that there’s a phase 1 study of a Bundibugyo virus vaccine. I guess it’s adenovirus-based [4]. How would you compare that platform with other vaccine platforms that might be available for an Ebola vaccine, both in terms of speed and effectiveness?

## TK

The short answer is that remains to be seen. That’s why you do the trials.

## GO

I think my point of view is every platform has a strength and a weakness. You’re always looking for the ability to get quick immune responses. In the case of the vesicular stomatitis virus (VSV) platform, which was used in Ebola Zaire, you can use it as a “ring vaccination”. There’s some debate about how that actually worked, but that is a possibility. Then you have durability, and you have safety in the process. So those three go back and forth, and you can vary each of these platforms. For example, with messenger RNA, you can look at the adenovirus systems, VSV, and some of the others being proposed; these all have issues, whether they have a lack of durability or an early immune response, and also a potential safety issue. So there are always strengths and weaknesses that have to be weighed. And you hope to hit it right on the head. These are expensive to develop. There’s no commercial use. So the government has to pay for these to be developed, and you’re talking an excess of $1 billion each time you make one of these vaccines.

So we have two vaccines for Zaire. We have candidates for Sudan and for Marburg virus, the sister virus of Ebola Zaire. And they were used in outbreaks. So they actually have phase 1 and phase 2 data now [5, 6], and then Bundibugyo was next on the list. But it wasn’t the priority. And so we live in the world of feast and famine in our business. There is either nothing to fund that project or there’s a lot. And that’s what we’re going to experience now with Bundibugyo.

## MML

In talking about the vaccines that are out there, is there any reason to think or hope that there’s any level of cross-protection between the available vaccines that have worked for Ebola Zaire and Bundibugyo?

## TK

I’d say there’s some suggestion of cross-reactivity and perhaps cross-protection. A small primate study that was done [7]. It’s not terribly definitive, but I think they were considering doing a clinical trial since that’s a licensed product. And I guess we’ll see.

## GO

And there have been some clinicians who are reporting that they were vaccinated and they got sick and were in the ICU, but their colleagues who weren’t vaccinated had a worse outcome. Again, we have to do the epidemiology to better understand that. But I agree with Dr. Ksiazek. It’s an unknown. The viruses are very different. From an immunological point of view, this is something we’ve seen over and over. You can vaccinate for Zaire. You can challenge with Zaire, and you can come back with Marburg, and the animals don’t survive. So these are not simple. But I think with the technology we have today, we can have a pan-Filo.

## ALL

Gene, you direct the Galveston National Lab, and it’s a wonderful resource in the United States. What role does this play during these emerging outbreaks in Africa or other parts of the world? How do BSL-4 labs contribute, and how can you contribute alongside Tom? And if we look globally, are there other labs out there in other parts of the world that are also working in conjunction with your efforts to help identify and understand more about this outbreak?

## GO

I think in the last 4 or 5 years, we’ve actually started to work together around the world in the sense that we’re trying to work on different targets and help each other. The BSL-4 labs have different capabilities. Some BSL-4 labs are simply diagnostic labs that don’t have the capability of doing any in vitro work. You have labs that can do in vitro, and you have labs that can do in vitro and animal studies. We have to use animal studies in these models to get licensure, because we hope we can succeed before we have outbreaks like this where we have enough patients to do a clinical trial. So the normal process is early basic research. Then we would develop prototypes and test those in different models, and finally move on to what we call nonclinical studies for the US Food and Drug Administration (FDA), which is the two-animal model in which we use a well-defined animal model that recapitulates the disease and allows us to license the product. That’s what was done with some hybrid work with human cases, with Zaire.

So that’s the process that we follow, and that’s what we’re working on. We’re well connected today. CDC has really been the key, and Tom can talk more about that, but they’ve been the key that links us all together. So, in normal situations, we’d all be working through them to do this work.

## TK

There’s still cooperation here in the US among the major labs, and there are links to other BSL-4 labs for in vitro work and, to a more limited extent, animal model testing. But I would say that the CDC has been somewhat hampered by a couple of circumstances. One is that the international organization that ties this all together and which has the charter for International Health is the WHO. WHO, in the World Health Assembly, is really the center for communicating the data from these outbreaks. The CDC is one of the arms that receives that information. And they contribute an awful lot in terms of epidemiology, laboratory diagnostics, and the whole gamut. However, that’s been cut off, and to a limited extent, many other US programs are also limited in their ability to communicate directly with the WHO.

So, for instance, a lot of the information I’m privy to doesn’t come from direct communications with those sources, but rather from press reports. And, that’s really unfortunate. I think the CDC is hindered to a certain extent in the same way. And then the other part of that is the cutbacks, and I would add, the politicization of the CDC hasn’t helped with their ability to freely participate in outbreaks like this now. So, the composite of those effects has, I think, limited the degree to which the US can play, and has played in the past, a leadership role in responding to these outbreaks.

## GO

People think of BSL-4 labs as these places where we play with dangerous viruses, and we store them. In reality, it’s an integrated research ecosystem. We actually have the capability of helping with diagnostics, vaccine evaluation, therapeutic testing, animal models, assay development, and reagent production, which Dr. Ksiazek is famous for doing.

We’re training the next generation of scientists. We’re training our replacements, and then we’re part of an international network. We connect internationally with other labs to help each other. So it’s way more complicated than just saying we’re all individual labs. Our sister lab is the Boston NEIDL, the National Emerging Infectious Disease Lab, and we have worked hand in hand on this outbreak. One of the key things we want to do is make sure we’re not duplicating work, and that we get the best work done at the right time and in the best location.

I think about this problem a lot. It’s like being a juggler. If you look at the select agent list, we have about 63 toxins and pathogens that are on that list. And we’re asking these labs — the BSL-2, BSL-3, and BSL-4 labs — to juggle these on a regular basis, and you’ve got to keep them going, but funding comes and goes. When one focus becomes important, you start prioritizing, and the challenge for us is: how do you manage the others at the same time? So we have ongoing work with other viruses — Nipah, Hendra — and how do you manage that during a crisis like this and keep everything moving, but paying more attention to one. So our real challenge is that we’re professional jugglers.

## TK

I can speak to CDC’s role. Their primary role, was to respond by putting people in boots on the ground during the outbreak. So priorities shifted pretty quickly during an exercise like that, where, as Gene mentioned, they did basic science and applied science on a regular basis when there wasn’t an outbreak. But, you have to put that in abeyance at least during an outbreak.

## ALL

And, Gene, are there any BSL-4 labs in the region that can help, and can you collaborate with local labs?

## GO

There is a lab in South Africa, and there’s also one in Gabon. I don’t know if the one in Gabon is actually functional at the moment, but the South African lab stays pretty busy, too. Dr. Ksiazek probably knows more about it. I’ve never visited that one.

## MML

These sophisticated laboratory facilities are really essential, and it’s critical that we keep them going, particularly in an era when there is some anxiety about support for science on a national as well as international level. But, when a person with Bundibugyo infection gets hospitalized, what are the containment practices that are necessary to provide care to that person, as well as to assure that the laboratories that are being used to evaluate that person’s course are managed safely?

## GO

I think Dr. Ksiazek has a wonderful explanation of this. We talk about countermeasures. We talk about diagnostics. But it gets down to epidemiology and basic case contact tracing.

## TK

They create these Ebola treatment centers during an outbreak where they’re not building or bringing in a BSL-4 environment. It’s largely based on the personal protective equipment (PPE) that can be used in facilities that are on the ground. There are some more bubble-like treatment units that are brought in, but historically it’s been based more on the PPE that the individuals providing treatment use. That has worked well. Another thing that I think a lot of people don’t recognize is that in an environment like that, in order to disinfect people, the use of chlorine bleach is very prominent. So when you approach an Ebola treatment center, you’re probably going to smell bleach. And that’s used to decontaminate the individuals and the environment on a regular basis, and it’s part of what makes it effective.

I might mention that we had a small outbreak here in the US when a patient came back to Dallas, Texas from Liberia. We got involved in that by disposing of a lot of the disposable PPE that was used in Dallas, because the normal channels wouldn’t accept it anymore. In that environment, there’s a heavy dependence on disposable PPE as opposed to in outbreak centers. Disinfection and the use of reusable gowns, etc., is one of the things that might be emphasized in a resource-poor environment, which these places certainly represent.

## MML

So, Tom, how do the clinical labs that are providing support for the hospitalized patient, how are they managed safely when samples are brought in for analysis?

## TK

It’s through a couple of means. Some people use small bubble-like environments to handle the materials. Others depend on gowning up and using PPE and breathing apparatus to protect themselves. But if you’re using molecular antigen detection assays, what’s done quickly is to inactivate the specimens you’re handling so they’re no longer dangerous to handle.

There’s usually some effort to put away viable or non-inactivated materials so they can be sent to other BSL-4 labs for further study. But the people who are in the field generally are working with material that they inactivate as a first step in handling them.

## MML

Yeah, I can see that this could be a huge challenge when a hospital is caring for a number of patients with an infection like this.

## GO

We’ve actually moved away from some of the assays we’ve had in the past. And what we have now is these different devices that are usually one patient per run, per sample, and that works really well when the numbers are small. But once you start hitting 100 cases a day, you have to move to other types of systems that are much more robust and actually require a lot more work.

So yeah, unless you have multiples of that piece of equipment-I won’t say the equipment, but there are multiple devices you can stack or use together to get more patients analyzed per day. Ultimately if you get over 100 patients, you need to be running the traditional extraction method then the traditional runs on these, and it takes time to do those. And for those, like Dr. Ksiazek said, we wear PPE that’s appropriate to make sure that we’re safe. But remember, a lot of times you’re doing this in a non-air-conditioned room. So it’s not like working in a lab at home. It’s very different.

## MML

I should think it’s kind of frightening when you think of the need to monitor electrolytes and fluids and chemistries for people who are desperately ill in a critical care setting, I should think it’s quite a challenge.

## TK

The really dangerous part of this is for the people who actually provide the clinical care. Incidents happen. And for instance, in the 2014 outbreak, the tales were that when this gets introduced into a clinical facility, in the end, the entire nursing staff gets wiped out by the disease. That’s unfortunate. Outsiders who come in and bring a lot of the PPE resources, etc., and also strengthen the infection control methods are a godsend and pretty necessary in these sort of environments. And this one is so wide in scope. How do you manage that? It’s a real difficulty. I would say agencies like Médecins Sans Frontières play a very large role because they have a lot of capacity, and they have the logistical umph to bring a number of these centers online with skilled personnel and routines that’ll be very important in operating a number of centers. But I would say at this point, it’s beyond the scope of what even they can handle.

## ALL

Tom, you were involved in identifying the first Bundibugyo outbreak in 2007. There’s been probably a lot of scientific advancement since then. What has improved our abilities now to respond better to filo outbreaks like this new one here in 2026? And what are the real gaps that still exist? What else do we really need to do, and how are we going to fill those gaps so it doesn’t happen again?

## TK

As Gene has already mentioned, the type of diagnostics that can be used have vastly improved. And we knew about this virus, and so putting together molecular assays at least is pretty simple. The way Bundibugyo was identified was that we had an antigen detection test, which was actually developed when the Reston outbreak occurred in suburban Washington, D.C., with monoclones that were made against both Zaire and Sudan when they were the only Ebola viruses that we knew about. Luckily, some of those monoclonal antibodies, because they were sort of selected to be among the ones that were cross reactive between the two viruses turned out to be cross reactive with Taï Forest, or Ivory Coast, as well as the Bundibugyo virus.

So the first hint that we had an Ebola virus on our hands at CDC, when specimens were sent from the Bundibugyo outbreak, was the antigen detection hit and the molecular assays that we had thought were cross-reactive because they would react with both Ebola Sudan and Ebola Zaire did not hit. And so as soon as we saw very strong positives in the antigen detection test and no positive results for the molecular assays, we kind of put our heads together and said, “Hey, we’ve got something here that is new.” We could at that point say it was an Ebola. And so we went back to Uganda with that information and the outbreak continued under the premise that it was an Ebola virus. Since then, again, the molecular assays have been developed that do have the breadth. And as Gene mentioned, the problem is getting as close as possible to the patients so that early rapid diagnosis can be done. And that can be reflected in the clinical setting and the epidemiologic setting.

## GO

One of the issues with the diagnostics, too, is that, in-country, we have licensed diagnostics for Ebola Zaire, so people will get the Ebola Zaire test. They’ll run it, and it’s negative. And they say, “Woo, it wasn’t Ebola Zaire.” They go on and assume it’s malaria or something else causing the issue. Whereas we all know in this field that we have these other Ebola viruses. We have Marburg that looks the same. But just having one and saying it’s not Ebola is not enough.

I’ve been screaming this for years with the different groups in Africa: we need to make sure we have all the different assays and they should be pan. They should be on one system that tells you what it is. A lot of the work right now is going into these molecular assays that give us each of those virus isolates.

## ALL

What are the gaps we really need to fill, especially locally? I mean we’re able to do a lot of advanced work here in the US. But what happens at the site because this is really where the rubber hits the road in this outbreak. How are we going to deal with this locally, and how do we improve our abilities of working in the field more? What are your thoughts on that?

## GO

Good question. I’ll start with a positive thing. In the US, we have assays. So if you went to the hospital, you would have those pan assays that would tell us if we have Marburg, Ebola Zaire, Sudan, and of course, Bundibugyo. We have those assays, and they can be used. We actually have the ability to handle patients. We have regions in the country here that can handle patients. We have one here at UTMB. We can handle patients and deal with treating them and give them really good care, even to the point of extracorporeal membrane oxygenation (ECMO) capability, which is something we don’t have in other parts of the world. So out in the field, we need something that’s rugged, that can last. We need to be able to move it quickly and be useable with a low level of learning required. So that’s why the LFA and those type of assays are so interesting.

I will say the molecular assays that are deployable, that are small, are getting that way. They’re getting to the point that you can run it and it’s pretty safe. But it also is electronic, so it has issues with failure. There are things we need to work on with that. And also speed. Some of them take 15 minutes. Some of them take 30 minutes or an hour or, so we need those kinds of things to change. But they have to be portable.

Michael, you brought this up earlier. In the clinics, they have used i-STAT and different types of Piccolo systems so they can get answers quickly while they’re standing there. One of the most important clinical tests they’re using is just diabetes, your sugar test, and seeing what your sugar levels are. That’s been huge for saving people as well, because they can monitor that and actually come back with interventions. So standard of care is really important, too, and we’re learning how to do that on that side.

## ALL

Gene, what’s throughput? I mean, if we’re looking at screening out in the field, how many samples are we looking at having to screen in a day? And how do these clinics handle all that?

## GO

In my experience in Liberia and Sierra Leone, we had the ability to do 96-well plates. Somebody could run those. On a daily basis, we ran somewhere between 70 and 80 samples. But that’s quite a bit of work. And we actually put the facilities right next to the Ebola Treatment Units (ETU). In Liberia, they had to bring them to us. It could be 4 hours before the sample got to us and we ran it; it could be the next day.

It’s important to have the assay right then, right there. Because when people walk in, you don’t know if they have malaria, you don’t know if they have Ebola, or in this case, in Uganda recently, they had Marburg. So how do you know what the disease is? Well, you can kind of make that decision quickly, and then you can triage people and put them in the Ebola unit and not potentially infect someone who’s not infected. So that’s an important part of this as well.

## MML

Artificial intelligence (AI) is making its way into our lives and into our medicine. How do you see AI helping us with preparedness, diagnostics, vaccine development, and therapeutics? What are you thinking about?

## GO

Well, I think AI has potential. We’re too early in the curve of understanding what we can do with it at the moment. One thing I’m really excited about is that, with this technology, discovery isn’t sequential anymore. We can actually learn from the past in the present and we can actually project outwards. So that’s going to be an important part of what we do. But how AI is going to make a difference in the eastern part of DRC right now? Probably not much. And that’s going to be hard to do in this round. But I think long term it can allow us to make vaccines that are pan Ebola. There are a couple of projects right now using AI to simulate different antigens that will give us some immune responses. And it’s using that capability to pull that off. That’s something we couldn’t have done before other than empirically, which is years of work.

## TK

I think another thing that it might help with if we get the right kind of point-of-care diagnostic test together, and that is: how do you compile those results so that you can use them in an epidemiologic sense, and putting together a network of cell phones that contribute to a database that allows the national authorities to know where this is and to know where it’s spreading to, is a very important thing that’s been lacking in the past, and it can probably help. The programming capabilities of AI should speed this greatly, and I would hope it does get exploited in the face of an outbreak like this.

## MML

So you think this could be really useful for tracking.

## TK

Yeah. It’s not just that I’m sitting here and I know this guy’s a patient, but rather now contributing to some sort of compiled data set where they’re allowed to see the big picture of what’s going on in the outbreak is, I think, an important aspect of this.

## GO

This goes all the way back to John Snow and being able to put it on a map and map it out. Being able to put cases where cases are occurring is important.

## ALL

Gene, the human element: We know a lot about the virus. We know a lot about virology and immunology, and the tools we have in diagnostics, therapeutics, and vaccines. But what are the operational and social factors? What about the human element? How do we get people to work in these environments, in what we’re dealing with here in the US, and over in Africa? How do we now really work more with people and their abilities to get involved? You mentioned during funerals that the people whose loved ones have died may go back and open the coffin and be exposed. And so how do we change human behavior to a point where people recognize this within the field? And then how do we also help our health care workers, increase their engagement and their abilities, and really motivate the unselfishness of people going there and actually exposing themselves to this deadly situation? How do we deal with the human element?

## GO

That’s a tough area. I definitely want Dr. Ksiazek’s points on this too. The first thing we need to do is protect our health care workers in that area, and I mean the national health care workers, the local health care workers, because, as Dr. Ksiazek says, these viruses basically just wipe out the health care system that’s already struggling to start with. So vaccinating those individuals before an outbreak could be very important, and during an outbreak could be important.

Trust is always an issue. It’s something that is best done in the country with people who know the culture. Our viewpoints don’t always make sense to them, and of course their points of view don’t make sense to us sometimes.

We need community engagement.

And one thing that was alluded to earlier is that we need security. We need to make sure the health care workers there are protected. You’ve seen a couple of cases where there’s been violence against either the burial groups or the hospitals. We need security around that. But we also need community engagement.

If you look at the West African outbreak, there was a lot of community engagement done that made a big difference. In this case, there’s essentially a civil war going on, and it’s very complicated. And so you’re trying to deal with an Ebola outbreak while they’re also dealing with malaria and their normal health care issues and a civil war. So it’s really hard. And you know this as well: Viruses don’t spread themselves. So how do you start breaking the chain of infection down? That’s done through very basic stuff. It’s not done through vaccines or therapeutics, typically. It’s done through case contact tracing and making sure we know where every case is coming from. And we don’t know that right now.

## TK

I would add that community engagement is terrifically important. One of the things that’s currently going on, and this is not new to this outbreak, is there’s a lot of misinformation, suspicion, believing this is not a real thing and that it’s curses and other things that the local populations don’t understand. And they’re quite resistant to cooperating with and being part of the control efforts. That needs to be broken down.

Anthropologists can play a very important role in these outbreaks. They shouldn’t be there just trying to study local beliefs, but rather trying to break down the disbeliefs and somehow present that this is an infectious disease, and there are control measures that actually work, and getting the community engaged in utilizing those methods in order to bring the outbreak to a stop.

If you use the 2014 outbreak as an example, it’s my belief that the communities were eventually what brought that outbreak to cessation. I think that in Liberia in particular, I can remember talking to some of my colleagues who were on the ground, and I think that they reached the point where they thought it was hopeless. In the end, it wasn’t hopeless. The projections that were initially made based on models really projected dire consequences, even worse than the actual outcome. I think, eventually, it gets so bad that the communities themselves begin to realize this is real, and they have to engage in some sort of control practices that make sense to stop the chain of transmission.

## GO

Radio is really important for them. Posters and signs make a big difference. We had the same issues with COVID-19. This is not just an African, eastern DRC problem. This is a problem around the world, and we have a lot of things that are fighting against us, social media and other things that will spread that kind of misinformation. And again, people transmit viruses and bacteria. They don’t just bop around by themselves, so we need to make sure that that’s understood.

## TK

COVID-19 was horrible. It’s a respiratory disease and it is transmitted by a completely different means than Ebola outbreaks, and as a bloodborne pathogen, the hope for being able to control Ebola is simpler. But it’s complicated by the social beliefs and misinformation that exist during outbreaks. But once people begin to realize that fairly simple means will help to control this, then I think the reality is that you can bring one of these outbreaks to cessation. It does take outside help, it takes resources. But if you use those in the right sequence and in the right places, you can defeat this.

## ALL

So, Tom, that’s an important point. What are some specific steps and who should take them with the current outbreak, because it’s growing. It’s not stopping. Its ongoing. What do we need to do today to really halt the current outbreak in terms of what’s going on with the Bundibugyo outbreak?

## TK

Well, more resources. There have been posts from WHO, from Africa CDC, requesting more monetary resources to supplement the communications aspect of this outbreak, and that is trying to make sure that the local people appreciate what this situation is. That requires getting skilled people in place who understand the culture, who speak at least the lingua franca for the country, French, etc. I think we’re behind the curve still on this outbreak, and that’s why we’re continuing to see the expansion of it.

## MML

It sounds as though trust, community engagement, and resources are really critical right now. And so in the future. How should we prepare for the next one? What are the research questions that have come up in recent outbreaks that we really need to prioritize over the next year or two?

## TK

I’d say early diagnosis is the first one because it’s fairly easy to control these outbreaks if you identify them very early when you’ve got a case or two on your hands, and you can stop the transmission chain before it really gets going. There’s an excellent paper that describes how, in Uganda, after the Gulu outbreak, CDC installed a national diagnostic lab in Uganda that then managed to nip in the bud a number of Ebola Sudan and Marburg potential outbreaks by getting in early and using these control methods. I think Trevor Shoemaker, I think, is the first author on that paper [8].

## ALL

One of the critical features going forward is with our policy makers and funding agencies. If we really want to impact Bundibugyo and future outbreaks, what message, Gene, do we need to make to those folks? It’s not just here in the US, it’s the global WHO, it’s Africa CDC. We know very well I could be in the Democratic Republic of the Congo today and on a plane tomorrow and in China, or I could be in Chicago, or I could be in Kuala Lumpur, or anywhere in the world. We live in a global community, and we have to think about this globally. So, Gene, how can we deal with this? What’s your message to the folks that make policy and funding decisions?

## GO

The main thing is that preparedness is never built on an emergency. We respond in a reactive mode versus being proactive. The feast-or-famine funding that we’ve received over the years: it’s steady funding, the right amount of funding at the right time is important. Too much funding is not good either. I’ve always said that as well, but the right amount of money. Then, really thinking about how we’re going to manage these outbreaks, in the sense of what Dr. Ksiazek said, we have to have good epidemiology, good diagnostics. That effort will give us the best chance to stop things quicker. We then need vaccines that are effective. They should be pan-filo, for example, or cross reactive. We need treatments that are oral and IV as well. That could be antibodies or small molecules.

All of these require a long path to licensure; they’re expensive. So the US government is moving towards, which I agree with, more agnostic approaches, broad-spectrum. We’ve tried this before. We’ve gone through this multiple times. Some things work out, and some things don’t. So it’s another investment in this area.

And again preparedness is an insurance policy that you pay for and you hope you never need it. That’s an important way to think about this. We think about that for our cars, but we don’t think about that for our global health.

## TK

Supporting global health is ultimately to our benefit as a national security effort. There was an article in STAT pointing out that we need to treat this as a national security issue [9]. There’s ultimately a lot more money there than at NIH, for instance. NIH could be the avenue through which the funds are distributed, but the emphasis as a national security issue is an important aspect of trying to make preparedness a real thing.

We just need to get on our horses and get after this one.

## MML

Well, then, let’s saddle up, gentlemen. Thank you so much for spending this time with us.

## SUPPLEMENTARY VIDEO

Supplementary videos are available at the *Pathogens and Immunity* YouTube channel.
